# Coffee Consumption and Risk of Biliary Tract Cancers and Liver Cancer: A Dose–Response Meta-Analysis of Prospective Cohort Studies

**DOI:** 10.3390/nu9090950

**Published:** 2017-08-28

**Authors:** Justyna Godos, Agnieszka Micek, Marina Marranzano, Federico Salomone, Daniele Del Rio, Sumantra Ray

**Affiliations:** 1 Department of Medical and Surgical Sciences and Advanced Technologies “G.F. Ingrassia”, University of Catania, 95124 Catania, Italy; marranz@unict.it; 2 Department of Epidemiology and Population Studies, Jagiellonian University Medical College, 31-008 Krakow, Poland; agnieszka.micek@uj.edu.pl; 3 Division of Gastroenterology, Ospedale di Acireale, Azienda Sanitaria Provinciale di Catania, 95024 Catania, Italy; federicosalomone@rocketmail.com; 4 Department of Food and Drugs, University of Parma, 43121 Parma, Italy; daniele.delrio@unipr.it; 5 NNEdPro Global Centre for Nutrition and Health (Affiliated with: Cambridge University Health Partners and the British Dietetic Association), St John’s Innovation Centre, Cambridge CB4 0WS, UK; sumantra.ray@mrc-ewl-hnr.cam.ac.uk; 6 Medical Research Council (MRC) Human Nutrition Research Unit, Cambridge CB1 9NL, UK

**Keywords:** coffee, caffeine, gallbladder cancer, biliary tract cancer, liver cancer, hepatitis, meta-analysis, dose–response

## Abstract

Background: A meta-analysis was conducted to summarize the evidence from prospective cohort and case-control studies regarding the association between coffee intake and biliary tract cancer (BTC) and liver cancer risk. Methods: Eligible studies were identified by searches of PubMed and EMBASE databases from the earliest available online indexing year to March 2017. The dose–response relationship was assessed by a restricted cubic spline model and multivariate random-effect meta-regression. A stratified and subgroup analysis by smoking status and hepatitis was performed to identify potential confounding factors. Results: We identified five studies on BTC risk and 13 on liver cancer risk eligible for meta-analysis. A linear dose–response meta-analysis did not show a significant association between coffee consumption and BTC risk. However, there was evidence of inverse correlation between coffee consumption and liver cancer risk. The association was consistent throughout the various potential confounding factors explored including smoking status, hepatitis, etc. Increasing coffee consumption by one cup per day was associated with a 15% reduction in liver cancer risk (RR 0.85; 95% CI 0.82 to 0.88). Conclusions: The findings suggest that increased coffee consumption is associated with decreased risk of liver cancer, but not BTC.

## 1. Introduction

Coffee is one of the most consumed beverages worldwide and it has been associated with a number of benefits on human health including a decreased risk of all-cause, cardiovascular, and cancer mortality [1,2,3]. Coffee is composed of a variety of compounds, some of which have been reported to have an impact on liver health [4]. Caffeine, a major component in coffee has been proposed to exert anti-carcinogenic effects toward up-regulation of antioxidant-responsive element (ARE)-mediated signalling [5], while phenolic compounds in coffee have been shown to exert anti-oxidant and anti-inflammatory effects [6]. In addition, coffee diterpenes have potential anti-carcinogenic effects [6]. However, besides the contribution of individual compounds or groups of compounds to liver health, synergistic effects are also possible. 

Gallbladder (GB) cancer is a highly fatal malignancy with notable geographical variations and a higher incidence in women. The aetiology of biliary tract cancer (BTC), including GB cancer and bile duct cancer (BDC) is poorly understood. One of the main risk factors has been hypothesized to be prolonged exposure to gallstones associated with chronic inflammation [7], which may increase the risk of BTC [8]. It has also been suggested that dietary factors contributing to gallstone formation can play a role in aetiology of BTC. Caffeinated and decaffeinated coffee consumption stimulates cholecystokinin release, which in turn stimulates the smooth muscle of gallbladder, causing its contraction [9]. However, gallbladder contraction may cause pain/colics in patients with gallstones. Moreover, coffee intake decreases cholesterol crystallization in bile, preventing gallstone formation [10] and modulating inflammation associated with the presence of gallstones [7].

Among the main targets of the healthful effects of coffee, the liver in particular, appears to benefit from coffee consumption due to the improvement of lipid metabolism regulation and decreased rates of liver steatosis and non-alcoholic steatohepatitis, which in turn may decrease the risk of consequent inflammation and fibrosis [11]. A recent systematic review showed that coffee consumption was consistently associated with lower rates of chronic liver disease and cirrhosis [12]. Interestingly, several studies have examined the impact of coffee consumption on fibrosis severity in patients with chronic viral hepatitis and have shown decreased rates of liver disease progression in coffee drinkers [13]. Recently, meta-analyses on coffee consumption and liver cancer risk have been published [14,15], but they lacked data on BTC risk, dose–response analyses, or analysis of possible confounding factors. The aim of the present study was to perform a meta-analysis of observational studies on coffee consumption and liver and BTC cancers, including the dose–response relation while taking into account the role of several potential confounding factors.

## 2. Materials and Methods

Meta-Analysis of Observational Studies in Epidemiology (MOOSE) protocols were followed throughout design, execution, analysis and reporting of this meta-analysis (Table S1) [16].

### 2.1. Search Strategy

We conducted a comprehensive literature search using PubMed (http://www.ncbi.nlm.nih.gov/pubmed/) and EMBASE (http://www.embase.com/) databases from the earliest available online indexing year to March 2017, with English-language restriction. Search terms included the following: (coffee OR caffeine OR beverages) and (extrahepatic OR gallbladder OR biliary tract OR liver OR hepatocellular) and (cancer OR carcinoma OR neoplasm) (Table S2). Two authors separately screened and retrieved the studies. We included prospective and case-control studies that evaluated association between dietary coffee intake and risk of extrahepatic/hepatic cancer in generally healthy adults. Studies were included if they provided corresponding risk estimates such as RRs (Risk Ratios), HRs (Hazard Ratios), or ORs (Odds Ratios). We excluded studies that reported insufficient statistics or insufficient coffee consumption categories (less than three; Table S3). Reference lists of included manuscripts were also examined for additional studies not previously identified. When duplicate publications from the same study were identified, we included the report that provided the largest number of cases/entire cohort or with the longest follow-up for each endpoint of interest. Full-texts of potentially relevant articles were assessed independently for eligibility by two authors.

### 2.2. Data Extraction

Data were abstracted from each identified study using a standardized extraction form. The following information was collected: (1) first author name; (2) year of publication; (3) study cohort name; (4) country; (5) number of participants; (6) sex of participants; (7) age range of the study population at baseline; (8) categories of consumption; (9) follow-up period; (10) endpoints and cases; (11) distributions of cases and person-years, HRs, and 95% CIs for all categories of exposure; and (12) covariates used in adjustments. This process was performed independently by two authors and discrepancies were discussed and resolved by consensus. The quality of included studies was assessed according to the Newcastle–Ottawa Quality Assessment Scale [17], which consists of three variables of quality as follows, selection (4 points), comparability (2 points), and outcome (3 points), for a total score of 9 points (9 representing the highest quality).

### 2.3. Statistical Analysis

In this meta-analysis, ORs and HRs were deemed equivalent to relative risks (RRs) [18]. ORs, RRs and HRs with 95% CI for all categories of exposure were extracted for the analysis and random-effects models were used to calculate pooled RR with 95% CI for the highest versus lowest category of exposure. The highest versus lowest analysis was performed to determine the relationship between coffee intake and risk of BTC and liver cancer. We included gallbladder cancer and extrahepatic/intrahepatic bile duct cancer in the same analysis, as their aetiology is similar [7]. The risk estimate from the most fully adjusted models in the analysis of the pooled RR was used. Heterogeneity was assessed using the *Q* test and *I*^2^ statistic. The level of significance equal to 0.10 was used for the *Q* test. The *I*^2^ statistic represented the amount of total variation that could be attributed to heterogeneity. *I*^2^ values ≤25%, 25–50%, 50–75%, and >75% indicated no, small, moderate, and significant heterogeneity, respectively. A sensitivity analysis by exclusion of one study at a time was performed to evaluate the stability of results and potential sources of heterogeneity. Subgroup analysis was only performed for liver cancer risk, in order to check for potential source of heterogeneity according to study design, gender and geographical area. To test for potential confounders/effect modifiers, subgroup analyses were performed according to smoking status, coffee type and hepatitis. Publication bias was evaluated by a visual investigation of funnel plots for potential asymmetry.

A dose–response analysis was performed using the method of Greenland and Longnecker to calculate study-specific liner and non-linear trend (generalized least-squares, GLS) based on results across categories of coffee intake [19,20]. Data were extracted on the level of coffee intake, distributions of cases and person-years (when available), and ORs/RRs/HRs with 95% CIs for ≥3 exposure categories. The median or mean intake of coffee in each category was assigned to the corresponding OR/RR/HR with the 95% CI for each study. When coffee consumption was reported in a range of intake, the midpoint of the range was used. When the highest category was open ended, we assumed the width of the category to be the same as the adjacent category. When the lowest category was open ended, we set the lower boundary to zero. Two-stage random-effects dose–response meta-analysis was performed to examine linear and non-linear relationship between coffee intake and risk of biliary tract cancer and liver cancer. In the first stage the method of Greenland and Longnecker (generalized least-squares, GLS) was used to calculate study-specific coefficients on the basis of results across categories of coffee intake taking into account the correlation within each set of retrieved ORs/RRs/HRs [19,20]. Non-linear dose–response analysis was modelled using restricted cubic splines with 3 knots at fixed percentiles (25%, 50%, and 75%) of the distribution [21]. The coefficients that had been estimated within each study by performing random-effects meta-analysis were combined. In linear dose–response meta-analysis the method of DerSimonian and Laird was used and in non-linear dose–response meta-analysis the multivariate extension of the method of moments was used to estimate the relative risks. We calculated an overall *P*-value by testing that the 2 regression coefficients were simultaneously equal to zero. We then calculated a *P*-value for non-linearity by testing that the coefficient of the second spline was equal to zero. All analyses were performed with R software Version 3.0.3, using dosresmeta and mvmeta packages (Development Core Team, Vienna, Austria).

## 3. Results

### 3.1. Study Characteristics

**Figure 1:**
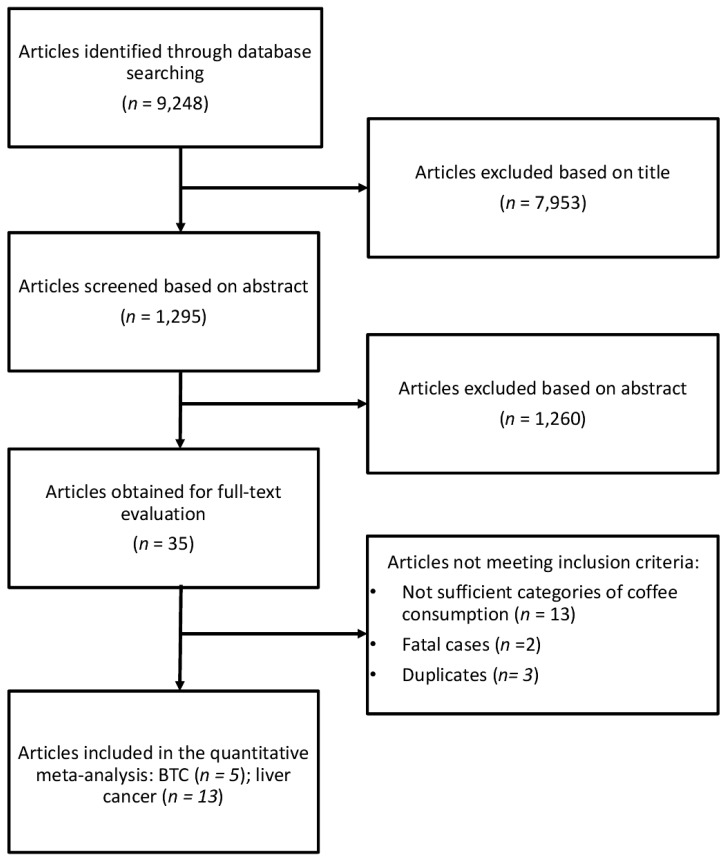
Flow chart and process selection of relevant studies exploring the association between coffee consumption and BTC and liver cancer risk.

The study selection process of eligible studies is presented in Figure 1. For the analysis on the association between coffee consumption and BTC risk five studies were eligible [22,23,24,25,26], one of which was a pooling project of nine cohort studies [25], two were studies comprising three prospective cohorts [23,24], and two were case-control studies [22,26]. Eligible studies included 1,375,626 participants and 726 BTC cases. The main characteristics of the studies included in the meta-analysis are summarized in Table 1. Six studies provided data for men and women separately [25,27,28,29,30,31]. Four studies provided data on type of coffee consumed [25,32,33,34], six on smoking status [25,27,28,30,31,35], and six on hepatitis [27,29,30,33,36,37]. Three studies were conducted in USA [22,25,26], one in Europe [23] and one in Asia [24]. The follow-up in prospective cohort studies ranged from about 13 to 20 years, and the age range at study baseline was 30–84 years.

Thirteen studies [25,27,28,29,30,31,32,33,34,35,36,37,38], including seven studies on six prospective cohorts and one multicentre study (EPIC) [28,29,30,31,32,34,38], one pooling project of nine prospective cohorts [25], and five case-control studies [27,33,35,36,37], were eligible for the analysis on the association between coffee consumption and liver cancer. Eligible studies included 2,105,104 individuals and 4227 liver cancer cases. The main characteristics of the studies included in the meta-analysis are summarized in Table 1. Two studies were conducted in USA [25,34], six in Europe [27,28,31,32,33,36], and five in Asia [29,30,35,37,38]. The follow-up in prospective cohort studies ranged from about 9 to 19 years, and the age range at study baseline was 20–79 years.

### 3.2. Summary Relative Risk for the Highest versus Lowest Category of Coffee Consumption

**Figure 2:**
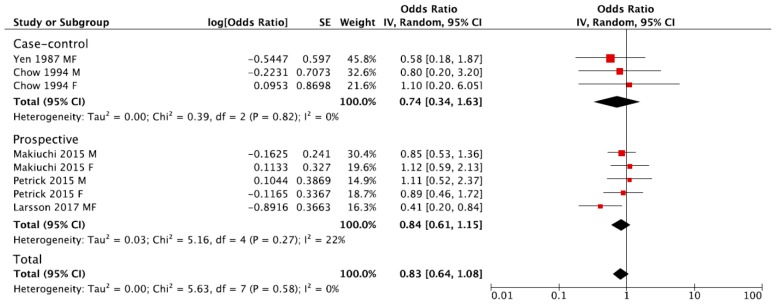
Forest plot of summary relative risks (RRs) of BTC for the highest versus lowest (reference) category of coffee consumption, by study design.

The summary RR of BTC for the highest versus lowest category of coffee consumption was 0.83, 95% CI: 0.64, 1.08, with no evidence of heterogeneity *I*^2^ = 0%, *p* = 0.58 (Figure 2). No publication bias was found after visual inspection of funnel plot (Figure S1). The pooled estimations were RR = 0.84, 95% CI: 0.61, 1.15; *I*^2^ = 22%, *p* = 0.27 for prospective cohort studies, and RR = 0.74, 95% CI: 0.34, 1.63; *I*^2^ = 0%, *p* = 0.82 for case-control studies (Figure 2). The subgroup analysis was not performed due to the limited number of studies eligible for the meta-analysis.

**Figure 3:**
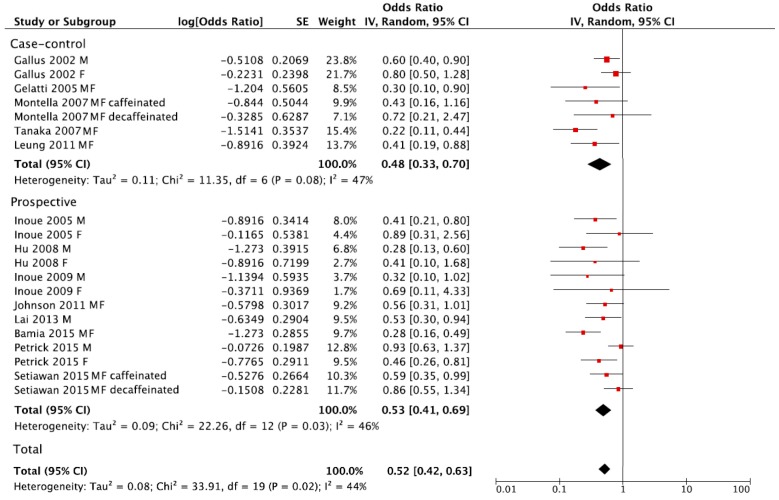
Forest plot of summary relative risks (RRs) of liver cancer for the highest versus lowest (reference) category of coffee consumption, by study design.

The summary RR of liver cancer for the highest versus lowest category of coffee consumption was RR = 0.52, 95% CI: 0.42, 0.63 with moderate heterogeneity *I*^2^ = 44%, *p* = 0.02, (Figure 3). However, no publication bias was found after visual inspection of funnel plot (Figure S2). The summary RR in separate analysis for prospective cohort studies was RR = 0.53, 95% CI: 0.41, 0.69; *I*^2^ = 46%, *p* = 0.03, and RR = 0.48, 95% CI: 0.33, 0.70; *I*^2^ = 47%, *p* = 0.08 for case-control studies (Figure 3). 

When considering sex and smoking status, no significant differences in comparison to main analysis of prospective cohorts were found (Table 2). In contrast, a significant decrease in risk of liver cancer for caffeinated coffee (RR = 0.65, 95% CI: 0.49, 0.86; *I*^2^ = 0%, *p* = 0.59), but not for decaffeinated (RR = 0.85, 95% CI: 0.63, 1.14; *I*^2^ = 0%, *p* = 0.96) was found (Table 2). In the stratified analysis, a lower risk of liver cancer was found among studies conducted in European and Asian countries compared to USA, even though all results were statistically significant (Table 2). Finally, stratified analysis by chronic hepatitis status did not significantly alter the results (Table 2).

### 3.3. Dose–Response Meta-Analysis

**Figure 4:**
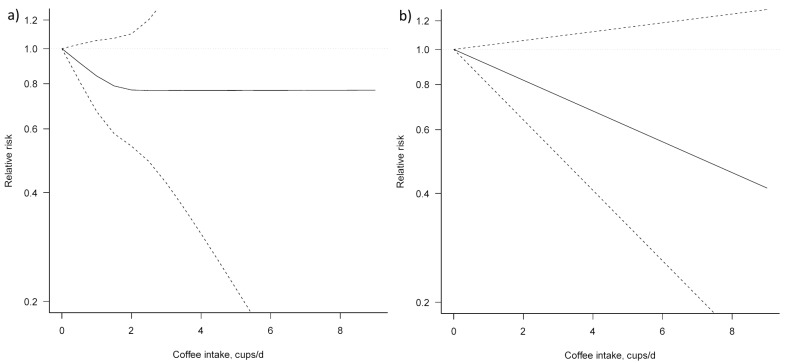
Dose–response association between coffee consumption and BTC risk (**a**) non-linear; (**b**) linear. Solid lines represent relative risk, dashed lines represent 95% confidence intervals.

Three studies [23,24,25] were eligible for dose–response meta-analysis of prospective cohort studies on coffee consumption and BTC risk. In both non-linear and linear dose–response meta-analysis no significant association between coffee consumption and BTC risk was apparent (Figure 4, Table 3). 

**Figure 5:**
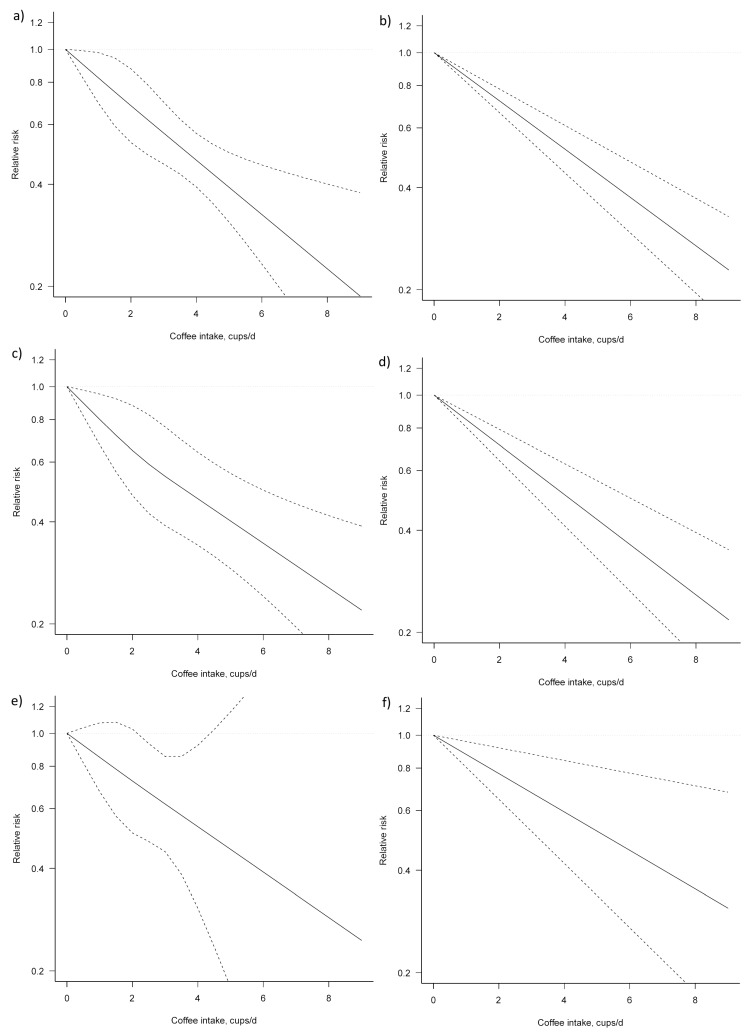
Dose–response association between coffee consumption and liver cancer risk: (**a**) non-linear, total analysis; (**b**) linear, total analysis; (**c**) non-linear, male; (**d**) linear, male; (**e**) non-linear, female; and (**f**) linear, female. Solid lines represent relative risk, while dashed lines represent 95% confidence intervals.

For the dose–response analysis on the association between coffee consumption and liver cancer risk, seven studies were eligible [25,28,29,30,32,34,38]. We found an evidence of linear association between coffee consumption and liver cancer risk (*P_for nonlinearity_* = 0.954) (Figure 5, Table 4). Compared with no coffee consumption, the pooled relative risks for liver cancer were: 0.82, 95% CI: 0.70, 0.98 for one cup/day; 0.68, 95% CI: 0.53, 0.88 for two cups/day; 0.57, 95% CI: 0.46, 0.70 for three cups/day; 0.47, 95% CI: 0.39, 0.56 for four cups/day; 0.39, 95% CI: 0.31, 0.50 for five cups/day; 0.33, 95% CI: 0.23, 0.46 for six cups/day; and 0.27, 95% CI: 0.17, 0.43 for seven cups/day. The associations were similar for men and women, although, in the analysis for women, a higher heterogeneity (*P_heterogeneity_* = 0.692) was observed.

## 4. Discussion

In the present meta-analysis, the inverse association between coffee consumption and risk of liver cancer was consistent when taking into account key potential confounding factors. In contrast, no significant association between coffee consumption and risk of BTC was evident. Notably, a non-significant decreased risk was found especially for lower intake of coffee (i.e., two cups/day); however, higher intake was associated with no further benefit or rather an increased risk in two out of the three cohorts examined. Furthermore, the limited number of the studies eligible for meta-analysis is not sufficient to draw conclusions on the association between coffee consumption and BTC risk. From a mechanistic point of view, intake of both caffeinated and decaffeinated coffee stimulates gallbladder contraction caused by increased concentration in plasma cholecystokinin induced by coffee and decreases gallbladder volume by approximately 30% [9]. Furthermore, coffee can exert a protective effect on gallbladder by decreasing the crystallization of cholesterol in bile [10]. However, induction of gallbladder contraction in patients with gallstones may induce the passage of gallstones to bile duct [7]. Overall, whilst a rationale for potential benefit exists, findings to date do not support such hypotheses. A possible reason for heterogeneity between results could depend on the different population involved that may have different health risk behaviours. For instance, higher intake of coffee was relatively poorly associated with alcohol consumption in the Northern European cohorts [39], which showed a decreased risk of BTC. In contrast, coffee was associated with higher alcohol intake in Asian [40] and US cohorts [41,42,43,44,45], which reported no benefits of coffee consumption on BTC risk. However, current data are not sufficient to reach final conclusions and further investigations are needed to clarify the relation between coffee consumption and BTC taking into account potential confounders.

Findings on coffee consumption and liver cancer risk were more consistent: all sensitivity and subgroup analyses performed showed significant decreasing risk of cancer with a linear dose–response relation. Molecular targets involved in the chemopreventive effects of coffee include the nuclear factor E2-related factor 2 (Nrf2), responsible for transcription of enzymes involved in detoxification processes and in cellular antioxidant defences [46]: a diet rich in coffee has been demonstrated to increase gene expression of NAD(P)H: quinone oxidoreductase 1, glutathione *S*-transferase class Alpha 1, UDP-glucuronosyl transferase 1A6, and the glutamate cysteine ligase catalytic subunit, all involved in the antioxidant response of the organism [47]. With special regard to hepatocellular carcinoma, coffee decreased the incidence of liver tumours in rats [48], reduced the numbers of hyperplastic liver cell foci in chemical models of colon and liver cancer [49], and reduced solid tumour growth, proliferation, and hepatoma metastases [50,51]. 

A number of experimental studies provided the biological rationale for the components responsible for beneficial effects of coffee on liver cells. In this meta-analysis, a significant decrease in risk of liver cancer for caffeinated coffee, but not for decaffeinated, was found. Caffeine has been reported to reduce fibrosis in in vitro and animal studies, inhibiting TGF-beta-induced CTGF (Connective Tissue Growth Factor) expression in hepatocytes by stimulation of degradation of the TGF-beta effector SMAD 2, inhibition of SMAD3 phosphorylation and up-regulation of the PPARgamma-receptor [52,53], as well as increased activity of superoxide dismutase and catalase in the liver and increased expression of Nrf2 [54]. 

It has been shown that the caffeine metabolite paraxanthine may be responsible for the down-regulation of the expression of the fibrogenic protein CTGF in hepatic stellate cells and reduction of liver fibrosis and lipid peroxidation [55]. More recent investigations have shown that caffeine is not essential for the anti-fibrotic effects of coffee. It has been demonstrated in animal studies that both caffeinated and decaffeinated coffee reduce liver fibrosis and TGF-beta expression [56,57] and that use of decaffeinated coffee is able itself to reduce liver steatosis, inflammation and fibrosis in animal models [58]. The phenolic compounds chlorogenic acids and caffeic acid are among the main candidates for the antioxidant effects of coffee on liver. Chlorogenic acids administration, or treatment in animal studies, reduces liver fibrosis through decreased expression of collagen I and collagen III, as well as reducing the expression of inflammatory cytokines, TLR4, myeloid differentiation factor 88, inducible nitric oxide synthase and cyclooxygenase-2 and nuclear factor-κB activation [59,60,61]. Caffeic acid reduces liver fibrosis due to its ability to suppress the activation of hepatic stellate cells by inhibiting oxidative stress through decrease of Keap1 expression, inhibition of Keap1 and Nrf2 binding, and thus activating Nrf2 and leading to increased expression of antioxidative signals [62,63,64]. Finally, coffee consumption may exert indirect protective effects on the liver due to the potential improvements of metabolism [3]. Coffee consumption has been inversely associated in several studies to metabolic syndrome [65,66,67,68,69,70,71,72,73,74], which has been related to liver fat accumulation and liver impairment due to common pathogenic determinants, such as insulin resistance and oxidative stress; impaired metabolism may induce progressive liver damage, liver inflammation and fibrosis, which ultimately may lead to carcinogenic transformation [75].

The results of the present study should be considered in the light of a number of limitations. First, some analyses reported moderate heterogeneity. As previously mentioned, several factors may explain differences across studies, including type of coffee bean (Arabica or Robusta), roasting, and beverage preparation. Secondly, genetic variants associated with caffeine metabolism are not considered in prospective cohort studies but were included in the meta-analysis and may contribute to the observed heterogeneity. Coffee consumption was assessed before outcome, thus recall bias is unlikely. However, misclassification of the actual amounts consumed may have affected the dose–response relation. Reverse causation may have affected the results if individuals changed coffee intake due to a diagnosed medical condition or disease; however, any such effects would be muted in studies with a long duration.

## 5. Conclusions

In conclusion, coffee may represent a valid functional food for liver protection. Current evidence is sufficient to guide future clinical randomized trials to test the hepatoprotective effects of coffee, which in turn may lead to more definitive recommendations. However, further observational studies with better in depth analyses of potential confounding factors are needed to test the association between coffee consumption and BTC.

## Figures and Tables

**Table 1:** Characteristics of the studies included in the meta-analysis.

Author, Year	Cohort Name, Country	Years of Study, Follow-up	Cases; Controls/Total Population	Age Range, Gender	Adjustments
**Biliary Tract Cancer**					
*Prospective*					
Petrick, 2015	LCPP, USA	Multicentre	260; 1,212,893	MF	Age, sex, race, cohort, BMI, smoking status, cigarette smoking intensity, alcohol.
Makiuchi, 2016	JPHC, Japan	1990–2010 Cohort I, 1993–2010 Cohort II, 20 years (maximum) Cohort I, 17 years (maximum) Cohort II	267; 89,555	40–69 years, MF	Age, sex, study area, BMI, history of cholelithiasis, history of diabetes mellitus, history of chronic hepatitis or cirrhosis, history of smoking, drinking frequency, physical activity by METs/day score, total energy consumption, energy-adjusted consumption of fish, red meat, and vegetable and fruit, and green tea.
Larsson, 2017	SMC, COSM, Sweden	1998–2012, 13.3 years	74; 72,680	45–83 years, MF	Age, sex, education, smoking, BMI, and diabetes.
*Case-Control*					
Yen, 1987	USA	1975–1979, NA	67/272	50–79 years, MF	Sex and age in decades.
Chow, 1994	USA	1985–1989, NA	98/226	30–84 years, MF	Age, ethnic origin, and smoking status.
**Liver Cancer**					
*Prospective*					
Inoue, 2005	JPHC, Japan	1990–2001 Cohort I, 1993–2001 Cohort II, 9.7 years (average)	334; 90,452	40–69 years, MF	Sex, age, study area, tobacco-smoking status, ethanol intake, green vegetable intake, green tea drinking.
Hu, 2008	Finland	1972–2002, 19.3 years (average)	128; 60,323	25–74 years, MF	Age, sex, study year, alcohol consumption, education, smoking, diabetes and chronic liver disease at baseline and during follow-up, and BMI.
Inoue, 2009	JPHC, Japan	1993–2006 Cohort II, 12.7 years (average)	110; 18,815	40–69 years, MF	Sex, age, area, smoking status, weekly ethanol intake, BMI, history of diabetes mellitus, green tea consumption, serum ALT level, HCV infection status, and HBV infection status.
Johnson, 2011	SCHS, China	1993–2006, 13 years (maximum)	362; 61,321	45–74 years, MF	Age at recruitment, gender, dialect group, year of recruitment, BMI, level of education, consumption of alcoholic beverages, cigarette smoking, frequency of black tea and green tea intake, history of diabetes.
Lai, 2013	ATBC, Finland	1994–2009, 18.2 years (median)	194; 27,037	~57 years (median), M	ATBC intervention arm, age, BMI, education, marital status, history of diabetes, years of smoking, cigarettes smoked per day, alcohol, tea intake, and serum cholesterol.
Bamia, 2015	EPIC, Multicentre Europe	1992–2010, 11 years (median)	201; 486,799	25–70 years, MF	Age at recruitment, centre, sex, diabetes mellitus, education, BMI, smoking, physical activity, alcohol intake, energy intake, simultaneously including tea.
Petrick, 2015	LCPP, USA	Consortium (AARP, AHS, USRT, PLCO, WHS, CPSII, IWHS, BWHS, WHI)	860; 1,212,893	MF	Sex, age, race, cohort, BMI, smoking status, cigarette smoking intensity, alcohol.
Setiawan, 2015	MEC, USA	1993–2010, 18 years (median)	451; 162,022	45–75 years, MF	Age, sex, and race/ethnicity, education, BMI, alcohol intake, smoking status, and diabetes.
*Case-Control*					
Gallus, 2002	Greece, Italy	I study (Italy) 1984–1997, II study (Greece) 1995–1998, NA	333/360 Greece; 501/1552 Italy	20–79 years, MF	Age, sex, smoking, tobacco smoking, alcohol drinking, BMI, history of diabetes and hepatitis.
Gelatti, 2005	Italy	1994–2003, NA	250/500	50–79 years, MF	HBV infection, HCV infection, alcohol intake, sex and age.
Montella, 2007	Italy	1999–2002, NA	185/412	43–84 years, MF	Gender, age, centre, education, smoking habits, maximal lifetime alcohol intake and serological evidence of HCV and/or HBV infection.
Tanaka, 2007	Japan	2001–2004, NA	209/1308	40–79 years, MF	Sex, age, heavy alcohol use and smoking status.
Leung, 2011	China	2007–2008, NA	109/125	MF	Age, gender, cigarette smoking, alcohol use, tea consumption, physical activity.

Abbreviations: AARP: (American Association of Retired Persons) Diet and Health Study; AHS: Agricultural Health Study; ATBC: Alpha-Tocopherol, Beta-Carotene Cancer Prevention ATBC Study; BWHS: Black Women’s Health Study; COSM: Cohort of Swedish Men; CPSII: Cancer Prevention Study II; EPIC: European Prospective Investigation into Cancer and Nutrition; IWHS: Iowa Women’s Health Study; JPHC: Japan Public Health Center-based Prospective Study; LCPP: Liver Cancer Pooling Project; MEC: Multiethnic Cohort Study; PLCO: Prostate, Lung, Colorectal, and Ovarian Cancer Screening Trial; SCHS: Singapore Chinese Health Study; SMC: The Swedish Mammography Cohort; USRT: U.S. Radiologic Technologists (USRT) Cohort; WHI: Women’s Health Initiative; WHS: Women’s Health Study.

**Table 2:** Subgroup analyses of studies reporting risk of liver cancer for the highest versus lowest (reference) category of coffee consumption.

Liver Cancer				
Subgroup	No. of Datasets	RR (95% CI)	*I*^2^	*P_heterogeneity_*
Total	20	0.52 (0.42, 0.63)	44%	0.02
Study design				
Prospective	13	0.53 (0.41, 0.69)	46%	0.03
Case-control	7	0.48 (0.33, 0.70)	47%	0.08
Gender				
Men				
Prospective	5	0.49 (0.30, 0.80)	64%	0.02
Case-control	1	0.60 (0.40, 0.80)	NA	NA
Women				
Prospective	4	0.53 (0.33, 0.83)	0%	0.71
Case-control	1	0.70 (0.50, 0.90)	NA	NA
Geographical location				
North America	4	0.72 (0.52, 0.98)	42%	0.16
Asia	7	0.42 (0.30, 0.58)	10%	0.35
Europe	9	0.48 (0.36, 0.64)	35%	0.14
Coffee type				
Caffeinated	3	0.65 (0.49, 0.86)	0%	0.59
Decaffeinated	4	0.85 (0.63, 1.14)	0%	0.96
Smoking status				
Never/former smoker	4	0.61 (0.43, 0.88)	32%	0.22
Current smoker	5	0.54 (0.36, 0.81)	61%	0.04
Chronic hepatitis				
Yes	7	0.56 (0.39, 0.80)	0%	0.87
No	5	0.60 (0.48, 0.75)	0%	0.71

**Table 3:** Dose–response meta-analysis of prospective cohort studies on coffee consumption and biliary tract cancer risk.

	No. of Datasets (No. of Studies)	Coffee Intake (Cups/Day)								*I*^2^ *(%)*	*P_heterogeneity_*	*P_non-linearity_*
		0	1	2	3	4	5	6	7			
Total analysis												
Non-linear	3 (3)	Reference	0.84 (0.67, 1.05)	0.77 (0.54, 1.10)	0.77 (0.43, 1.38)	0.77 (0.31, 1.91)	0.77 (0.22, 2.70)	0.77 (0.15, 3.86)	0.77 (0.11, 5.54)	0.54	0.15	0.46
Linear	3 (3)	Reference	0.91 (0.80, 1.03)	0.82 (0.64, 1.06)	0.75 (0.51, 1.09)	0.68 (0.41, 1.12)	0.61 (0.33, 1.15)	0.56 (0.26, 1.18)	0.50 (0.21, 1.22)	0.18	0.13	NA

**Table 4:** Dose–response meta-analysis of prospective cohort studies on coffee consumption and liver.

	No. of Datasets (No. of Studies)	Coffee Intake (Cups/Day)								*I*^2^ *(%)*	*P_heterogeneity_*	*P_non-linearity_*
		0	1	2	3	4	5	6	7			
Total analysis												
Non-linear	7 (6)	Reference	0.82 (0.70, 0.98)	0.68 (0.53, 0.88)	0.57 (0.46, 0.7)	0.47 (0.39, 0.56)	0.39 (0.31, 0.5)	0.33 (0.23, 0.46)	0.27 (0.17, 0.43)	54.18	0.010	0.954
Linear	7 (6)	Reference	0.85 (0.82, 0.88)	0.72 (0.66, 0.78)	0.61 (0.54, 0.69)	0.52 (0.44, 0.61)	0.44 (0.36, 0.54)	0.58 (0.34, 0.98)	0.32 (0.24, 0.42)	17.54	0.296	NA
Male												
Non-linear	5 (5)	Reference	0.73 (0.57, 0.94)	0.56 (0.36, 0.85)	0.47 (0.30, 0.72)	0.42 (0.29, 0.60)	0.38 (0.27, 0.53)	0.33 (0.23, 0.46)	0.30 (0.19, 0.48)	72.9	0.000	0.286
Linear	4 (4)	Reference	0.84 (0.80, 0.89)	0.71 (0.64, 0.79)	0.60 (0.51, 0.71)	0.51 (0.41, 0.63)	0.43 (0.33, 0.56)	0.55 (0.47, 0.63)	0.31 (0.21, 0.44)	15.48	0.314	NA
Female												
Non-linear	4 (4)	Reference	0.87 (0.72, 1.06)	0.76 (0.56, 1.03)	0.65 (0.46, 0.92)	0.56 (0.31, 1.01)	0.48 (0.19, 1.22)	0.32 (0.22, 0.49)	0.35 (0.06, 1.90)	0	0.586	0.938
Linear	3 (3)	Reference	0.88 (0.80, 0.96)	0.77 (0.65, 0.92)	0.68 (0.52, 0.88)	0.59 (0.42, 0.84)	0.52 (0.34, 0.81)	0.53 (0.44, 0.65)	0.40 (0.22, 0.74)	0	0.692	NA

## Supplementary Materials

The following are available online at www.mdpi.com/2072-6643/9/9/950/s1, Table S1: Meta-Analysis of Observational Studies in Epidemiology (MOOSE) checklist, Table S2: Search strategy, Table S3: Excluded studies, Figure S1: Funnel plot for BTC risk of the highest versus lowest (reference) category of coffee consumption, Figure S2: Funnel plot liver cancer risk of the highest versus lowest (reference) category of coffee consumption).
